# Structural Characteristics and Properties of Zinc Coatings on Steel Structural Elements

**DOI:** 10.3390/ma19132727

**Published:** 2026-06-25

**Authors:** Małgorzata Witkowska, Marcin Kowalski, Joanna Kowalska, Kinga Chronowska-Przywara

**Affiliations:** 1 Faculty of Metals Engineering and Industrial Computer Science, AGH University of Krakow, av. Mickiewicza 30, 30-059 Krakow, Poland; joannak@agh.edu.pl; 2 Institute of Technology, University of the National Education Commission, Podchorążych 2, 32-084 Krakow, Poland; 3 Faculty of Mechanical Engineering and Robotics, AGH University of Krakow, av. Mickiewicza 30, 30-059 Krakow, Poland; chronows@agh.edu.pl

**Keywords:** zinc coating, hot-dip galvanizing, adhesion, steel, microstructure

## Abstract

**Highlights:**

- Metallographic analysis revealed a multilayer structure composed of η, ζ, δ, and Γ phases with distinct chemical compositions.
- Hot dip zinc coatings produced on S235JR steel demonstrated good adhesion and structural stability.
- The study included metallographic, mechanical, diffraction, and tribological analyses, confirming the functional performance of the coatings.

**Abstract:**

This paper presents the structural characterization of zinc coatings on S235JR steel elements. The study offers a novel and comprehensive assessment of zinc coatings applied to profiled steel elements through hot-dip galvanizing. It examines coatings formed under real industrial production conditions, providing practical insight into their behavior on complex geometries. The characterization includes metallographic, mechanical, diffraction, and tribological tests. Metallographic observations revealed the layered structure of zinc coatings, consisting of the η, ζ, δ, and Γ phases, each with varying chemical compositions and microhardness. All coatings exhibited similar resistance to damage initiation; however, microscopic analysis revealed differences in their subsequent degradation. The thickest coating showed earlier formation of adhesive cracks, indicating increased stress concentration and a faster progression of damage.

## 1. Introduction

Carbon steels, despite their widespread use and favorable mechanical properties, are inherently susceptible to corrosion when exposed to atmospheric, industrial, or marine environments. Metal elements applied in the production of structures used in corrosive environments are subject to deterioration and even complete degradation over time. Various surface protection methods are used to ensure greater durability, with galvanizing considered one of the most effective and economical methods of protecting steel against corrosion and increasing resistance to mechanical damage.

Various galvanizing technologies enable coatings with different properties to be obtained. Hot-dip galvanizing is one of the most frequently used and most effective technologies [1,2,3,4,5,6,7,8,9,10,11,12,13,14,15,16,17,18,19,20,21,22,23,24,25,26,27,28]. The coating production process is not complicated, and the coatings themselves provide effective protection in various atmospheric environments for many years.

Obtaining such coatings involves the immersion of structural elements in a metal bath for a time sufficient to create a suitably thick coating [9,11,12]. This process leads to the formation of a multi-layer coating composed of various intermetallic phases based on iron and zinc, Fe–Zn (Γ, δ, ζ), and the outer layer η, consisting of practically pure zinc. Each of these layers is characterized by different physical, mechanical, and electrochemical properties, which give the zinc coating complex behavior under operating conditions [9,10,11,12,18,19,28,29,30,31,32,33].

The thickness and microstructure of these layers depend on a number of factors independent of the coating manufacturer, such as the grain size of the steel, its chemical composition, and the thickness and geometry of the components [34,35]. A separate group of factors influencing the coating thickness and structure that can be controlled includes bath temperature, its chemical composition [5,7,9,20,36,37,38,39], immersion time, and the speed of removal from the bath [5,7,9,11,13,16,39,40]. The coating thickness obtained by hot-dip galvanizing is between 70 and 150 μm.

During operations, steel elements covered with a zinc layer are exposed not only to corrosive factors but also to mechanical loads, including friction, erosion by solid particles, impacts, and abrasion [27,41]. Abrasion resistance is therefore a key parameter determining the durability of coatings, especially in applications such as road barriers, bridge structures, machine elements, and electricity pylons. The tribological properties of hot-dip galvanized coatings depend strongly on their microstructure. The soft η layer wears out quickly in the initial phase of abrasion, while the harder layers, including Γ, δ, and ζ, show much greater resistance to abrasive wear [42,43].

Literature research [7,12,40] indicates that element geometry may cause the formation of coatings with various thicknesses, discontinuities, or local overheating, which results from different dynamics of liquid zinc flow and variable intensity of diffusion reactions on the steel surface. Elements with complex shapes are more likely to form excessively thick intermetallic layers, which may be brittle and susceptible to mechanical damage. In turn, thin-walled or openwork structures may be exposed to insufficient coverage or the formation of defects related to rapid cooling. In industrial practice, this problem is particularly important because modern steel structures increasingly use elements with complex geometries, manufactured by welding, laser cutting, and bending methods. At the same time, requirements for operational durability are increasing, which requires a precise understanding of how the component shape affects the galvanizing process and the coating properties. Despite the large number of studies on the kinetics of zinc coating growth, relatively few analyze the relationship between element geometry and coating structure and quality, which justifies the need to address this topic.

Therefore, undertaking research into the influence of the shape of steel elements on the hot-dip galvanizing process and on the coating microstructure and properties is fully justified. The results of such research can help expand knowledge, improve coating quality, increase structural durability, and develop design guidelines for components intended for galvanizing.

The novelty of the research lies in the use of substrates that are not laboratory samples, but common structural components produced through various production processes. Although the grade of steel used as the substrate in each case is the same, the products’ properties, including their ability to form and grow coatings, vary, as demonstrated by the carried out metallography, tribology, and diffraction studies.

## 2. Materials and Methods

**Figure 1:**
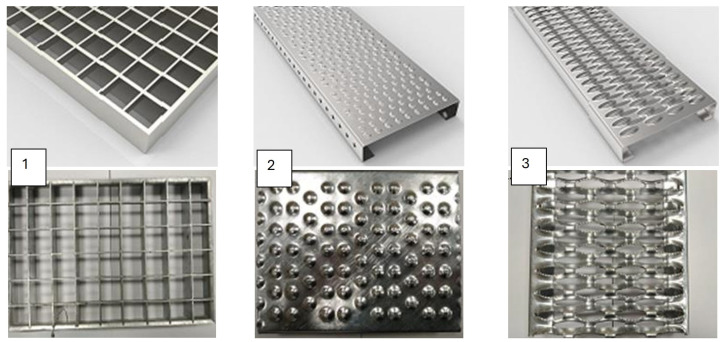
View of the profiles used for research.

The test material consisted of fragments of structural elements covered with a zinc coating (Figure 1). The elements were made of S235JR structural steel with the chemical composition shown in Table 1. Raw strip chemical composition analysis was performed using optical emission spectroscopy (OES) using a Foundry Master-WAS Spectrometer (Hitachi, Tokyo, Japan). The tested fragments were subjected to galvanizing under industrial conditions.

Preparing the elements for hot-dip galvanizing involved a conventional approach consisting of successive stages:

- Degreasing in an acidic solution,
- Digestion in an 18% HCl solution for 40 min,
- Rinsing in water,
- Flux treatment in a ZnCl_2_-NH_4_Cl solution at 40 °C for about 5 min,
- Drying at about 120 °C for 15 min,
- Immersion in a bath of molten zinc for 4 min; the bath temperature was 450 ± 5 °C, and its chemical composition is given in Table 2. These values were obtained from the manufacturing process.

Three different grating models, different in shape and manufacturing technology, were used for the tests. Two of them were made by embossing (numbers 1 and 2), while the third (number 3) was formed by pressing technology. Profile number 1 was cut and shaped appropriately, whereas profile 2 was characterized by round protrusions. In the case of profile number 3, the technology involved pressing load-bearing and transverse flat steel bars to create a grid. The profiles used for the research are shown in Figure 1.

**Figure 2:**
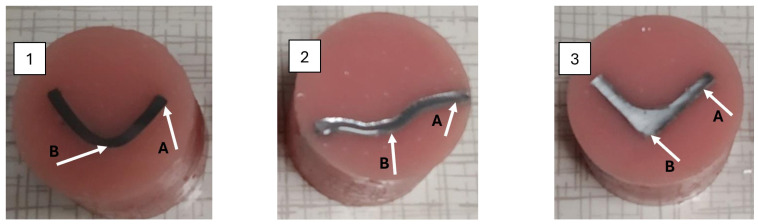
Galvanized steel samples used for testing: A—straight sections, B—embossed locations.

Microstructures were observed (Figure 2) using a Phenom XL (Thermo Fisher Scientific, Waltham, MA, USA) scanning electron microscope. In addition, EDS analysis of the chemical composition of individual coating layers was performed. In order to obtain contrast between particular structural components, chemical digestion was performed using a reagent consisting of 5 mL of nitric acid and 95 mL of alcohol (Nital). A microhardness test was performed on an Innovatest microhardness tester (Innovatest Europe BV, Maastricht, The Netherlands) using the Vickers hardness measurement method with a load of 10 gf (0.098 N) and measurement time of 10 s. The microhardness values are the average of 5 measurements for each layer (η, ξ, and δ), with the standard deviation being calculated accordingly.

Diffraction tests were performed with a Bruker Advance D8 (Bruker AXS GmbH, Karlsruhe, Germany) diffractometer using filtered radiation from a cobalt anode lamp (λ = 0.17902 nm) in the angular range of 2θ = 30 ÷ 65°. The phase analyses were performed using Bragg–Brentano (BB) geometry. The depth penetration of X-rays can be estimated using Formula (1) [44].

(1) $$X=\frac{-\ln\left(1-Gx\right)sin\theta }{2\mu }$$

where:

X—X-ray penetration depth
Gx—the intensity of the primary X-ray
μ—the linear absorption coefficient

A T-21 tribotester [45], produced at the Institute for Sustainable Technologies in Radom (Poland, Radom), was used to measure the coefficient of friction (CoF) and to determine the wear index (W_v_). Friction and wear tests were performed under concentrated ball-on-disk contact conditions. The accepted load of F_n_ was equal to 1 N, with the rotational speed n equal to 120 rev/min. Tests were performed for 2500 cycles using an Al_2_O_3_ ball with a friction tip radius R_t_ equal to 3 mm. The measured tangential force F_t_ was used to calculate the coefficient of friction (2) [46].

(2) $$CoF=\frac{F_{t}}{F_{n}}$$

**Figure 3:**
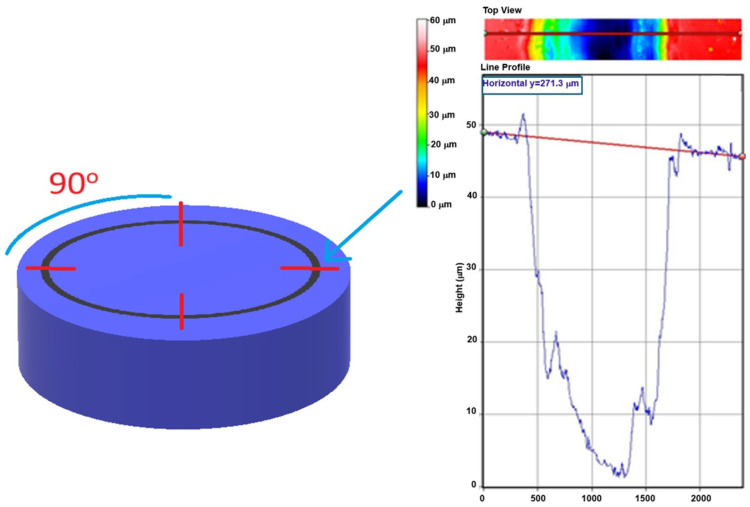
Measurement locations for the volume of cracks. Cross-sectional profile of the groove.

A Filmetrics 3D optical profilometer was used to measure wear marks. Material loss was calculated by determining the groove profile measured with the optical profilometer. Then, using the Gwyddion program (GNU General Public License, v.2.67, 2024, Czechia, Brno), the cross-sectional area of the groove was calculated in four places on the circumference, every 90°, taking into account the ratio of the outflow to the groove area, as in Figure 3. Figure 3 shows an image of the groove wear for one of the measurements, showing the measurement methodology. Next, the volumetric consumption rate was calculated with Equation (3) [47]. The tests were performed on three samples. Three tests were performed on each sample.

(3) $$W_{v}=\frac{V}{F_{n}\cdot n}\left[\frac{mm^{3}}{N\cdot m}\right]\;$$

where:

V—volume of the worn material [mm^3^];
F_n_—normal force applied to the sample [N];
n—friction path [m]

**Figure 4:**
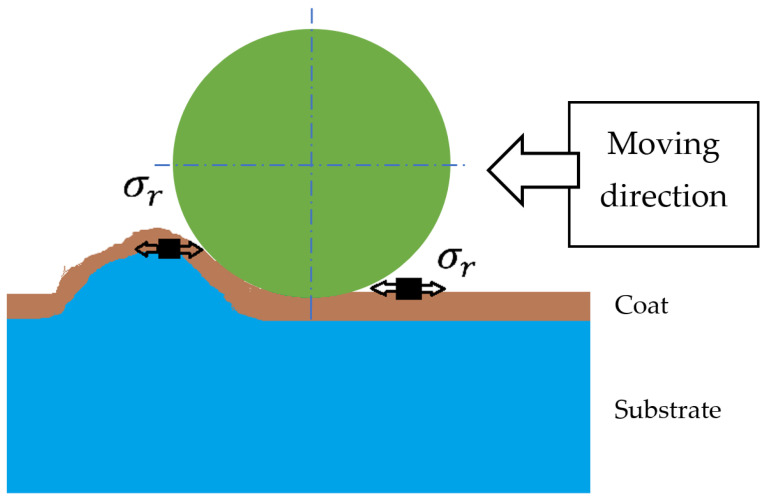
Concentration of tensile stresses in the coating.

Coatings perform their function when properly adhered to the substrate. Such coatings transfer the load during concentrated contact. The most common method for testing the adhesion strength of a coating to a substrate is the scratch test [48]. The test involves making a 3 mm long scratch with a Rockwell diamond indenter with a rounded tip radius of 200 µm under a normal load, F_n_, equal to 1 N. Increasing the load causes an increase in elastic and plastic deformations, leading to the formation of cracks (Figure 4). The maximum critical loads, Lc_,_ may correspond to cohesive cracks, Lc_1_, or adhesive ones, Lc_2_. Typical forms of cohesive failure appear as cracks perpendicular to the movement of the indenter. Adhesive cracks, on the other hand, appear as local delaminations at the outflow in front of the indenter and on the sides of the resulting crater. After exceeding the critical force, further adhesion cracks cause delamination of the coating from the substrate [49,50,51].

## 3. Results and Discussion

### 3.1. Microstructure and X-Ray Analysis

**Figure 5:**
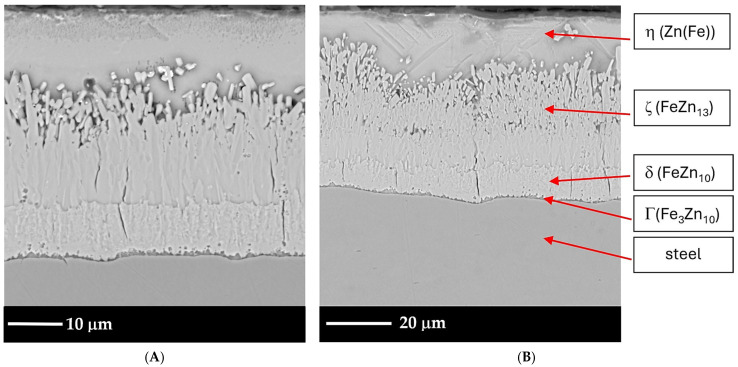
Microstructure of steel sample number 1, areas (**A**,**B**).

**Figure 6:**
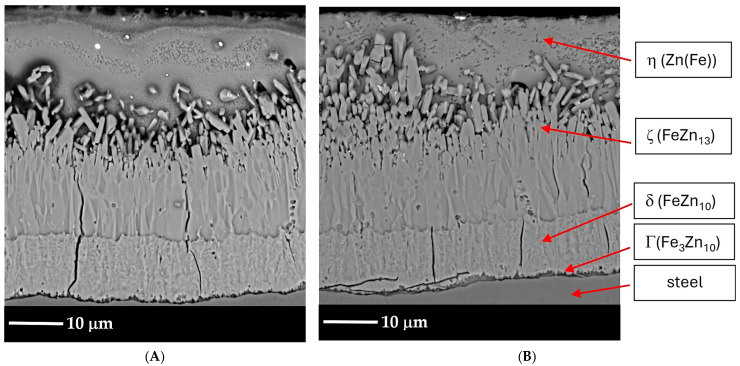
Microstructure of steel sample number 2, areas (**A**,**B**).

**Figure 7:**
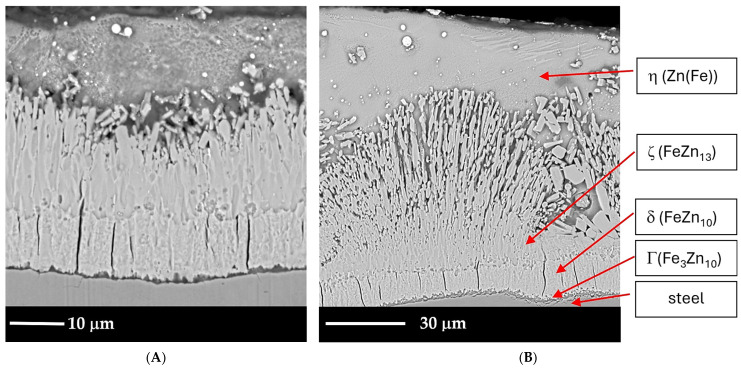
Microstructure of steel sample number 3, areas (**A**,**B**).

The results of metallographic investigations for individual samples are presented in Figure 5, Figure 6 and Figure 7. The tests were performed on the outer part of the straight sections (area A) of each sample, at the embossed location (area B) for samples 1 and 2, and at the corner of sample 3. In the microstructures of all samples, a layered zinc coating was observed, consistent with the literature [5,7,9,10,17]. The Γ (Fe_3_Zn_10_) phase layer was difficult to observe due to its thinness (less than 1 μm) and its direct crystallization on the substrate. Subsequently, the δ (FeZn_10_) phase layer was formed, and it was much thicker, having a relatively compact structure with densely packed columnar crystals. This layer was uniform in thickness, and perpendicular cracks were observed in it [2,9,13]. They were caused by tensile stresses that arose during cooling, resulting from the different thermal expansion coefficients of this phase and steel [52,53,54]. Cracks could occur during the preparation of the specimens. Similar cracks in the δ layer during solidification after hot-dip galvanizing were observed by Sirin [55]. The δ layer appeared to consist of two sublayers adjacent to the ζ (FeZn_13_) phase. The inner one had a more compact morphology and changed into a less homogeneous structure composed of loosely packed crystals arranged in the form of a “lawn”. The outer part of the coating consisted of a zinc η (Zn(Fe)) layer, which is a solid solution of iron in zinc. The microstructure showed continuity at the interface between the coating and substrate. Regardless of the manufacturing technologies used, the coatings obtained on the samples did not show significant differences in the microstructure morphology. The cross-section of each coating indicated the presence of successive Γ, δ, ζ, and η layers, which is consistent with the data published by Kania and Liberski [5,9,19]. Greater variation in the microstructure regarding the thickness of individual layers was observed between curved areas (B-embossed area/corner part) and straight areas (A).

Table 3 shows the chemical composition of individual coating layers. The test results represent the average of five measurements taken from each layer. The ζ layer contained approximately 93.5% Zn and 6.5% Fe for each sample. For the δ layer, zinc content ranged from 90.2 to 91.7%, and iron content ranged from 8.3 to 9.8%. The greatest differences in chemical composition occurred within the Γ layer, with zinc content ranging from 57.4 to 72.8 and iron content ranging from 27.2 to 42.6. Comparing the obtained results with the literature [19,56], a high correlation was observed in the chemical composition of individual layers, with the exception of the Γ layer. Large discrepancies in the chemical composition resulted from the fact that this layer was very thin, and the obtained results of the chemical composition analysis were influenced by the substrate. Depending on the manufacturing technology and the testing location (straight section (A) and curved embossed section (B) or corner section (B)), the thickness of the δ, ζ, and η layers changed. Table 4 shows the average thicknesses of the individual layers forming the coating on straight sections and at the embossed point (samples 1 and 2) and in the corner of sample 3. The widest layer was ζ. The total coating thickness at the test locations ranged between 39.8 μm and 53.4 μm. The exception was the coating thickness measured at the pressed part, which was twice the largest of the remaining results and equaled 95 μm. The reason for this difference was that the thickness of the iron solid solution in the zinc, i.e., the ζ phase, differed significantly from the thickness of this phase in other examined locations. The thickness of the ζ and δ phases ranged from 15.9 ÷ 53.7 and 7.9 ÷ 11.6 μm, respectively. The high coating thickness of sample 3B was attributed to the presence of sharp edges, over which zinc flowed more slowly. Zinc remained longer in hard-to-reach places before it drained, and local lumps could form [5,7,12].

**Figure 8:**
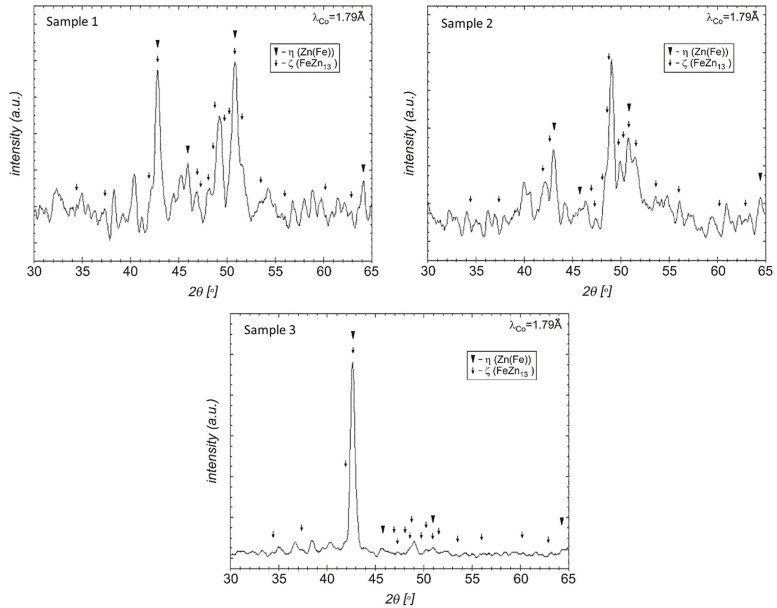
X-ray diffraction patterns of zinc coatings.

Figure 8 shows the X-ray diffraction patterns obtained for individual samples. The differentiation pattern showed peaks related to the η and ζ phases. The lack of lines from the δ and Γ phases results from the fact that the X-ray penetration depth was approx. 9–20 μm; however, the thickness of the coatings was greater. Therefore, there was no diffraction effect observed from the phases occurring in the deeper layers of the coating.

### 3.2. Microhardness Measurements

**Figure 9:**
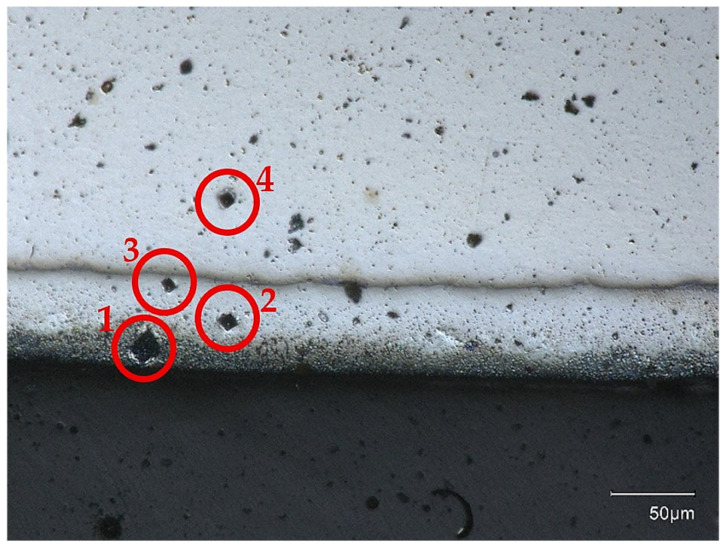
Example of hardness measurements indication position for sample 2. Number of indentations acc. to Table 5.

Table 5 shows the results of microhardness measurements. Measurements were made on a straight section of each sample and at the embossed point (samples 1 and 2) and the corner (sample 3). Four measurements were made at different distances from the surface. Figure 9 shows the microhardness measurements for sample 2; measurements were performed analogously for the remaining samples. The individual phases were distinguished based on their hardness values. As shown in Table 5, the η-phase exhibited the lowest hardness, ranging from 52–78 HV, while the ζ- and δ-phases were considerably harder, reaching 112–173 HV (ζ) and 230–280 HV (δ), respectively, which is consistent with the literature data [15,42,55]. Due to its very small thickness, the hardness of the Γ-phase could not be measured with the available testing method. Regarding the hardness of the steel substrate, higher values were observed, possibly due to solid solution hardening of the steel near the interface.

### 3.3. Tribological Measurements

**Figure 10:**
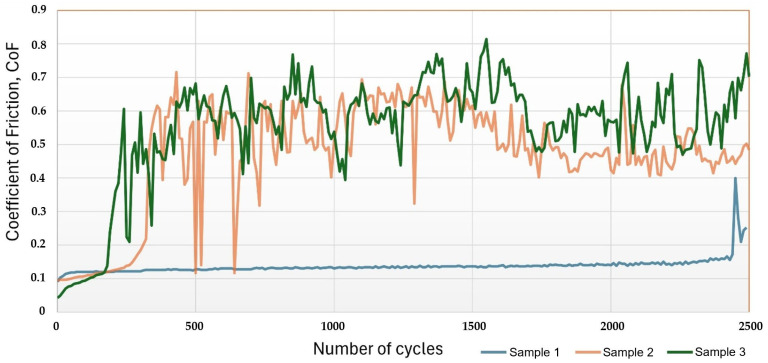
Coefficient of friction.

Figure 10 presents the evolution of the coefficient of friction (COF) as a function of sliding time for the investigated samples. Distinct differences in friction behavior were observed among the tested materials, indicating the significant influence of surface properties and wear mechanisms on the tribological response.

Sample 1 exhibited the lowest coefficient of friction throughout the entire test. Following a short running-in period, the COF stabilized at approximately 0.13–0.15 and remained nearly constant for most of the sliding duration. Such behavior indicates stable contact conditions and limited surface degradation. A sudden increase in the COF was observed near the end of the test, which may be associated with local damage to the surface layer or the progressive removal of a protective tribofilm.

In contrast, sample 3 showed the highest friction values, with the COF increasing rapidly during the running-in stage and subsequently fluctuating between approximately 0.55 and 0.75. The large variations observed during steady-state sliding suggest unstable contact conditions and the occurrence of intensive wear processes. Periodic increases and decreases in the COF may be related to the formation and removal of wear debris within the contact zone.

Sample 2 demonstrated intermediate tribological performance. After the running-in period, the COF stabilized within the range of approximately 0.45–0.65, which was lower than that observed for sample 3. However, several abrupt decreases in friction were recorded during the test. These transient events may indicate temporary changes in the contact configuration, local formation of transfer layers, or variations in the amount of wear particles entrapped within the sliding interface.

The results indicate that sample 1 provided the most favorable tribological performance, characterized by the lowest and most stable coefficient of friction. Sample 2 exhibited moderate friction, whereas sample 3 exhibited the highest friction and greatest instability during sliding. These findings suggest substantial differences in the wear mechanisms and surface interactions occurring in the investigated material systems.

**Figure 11:**
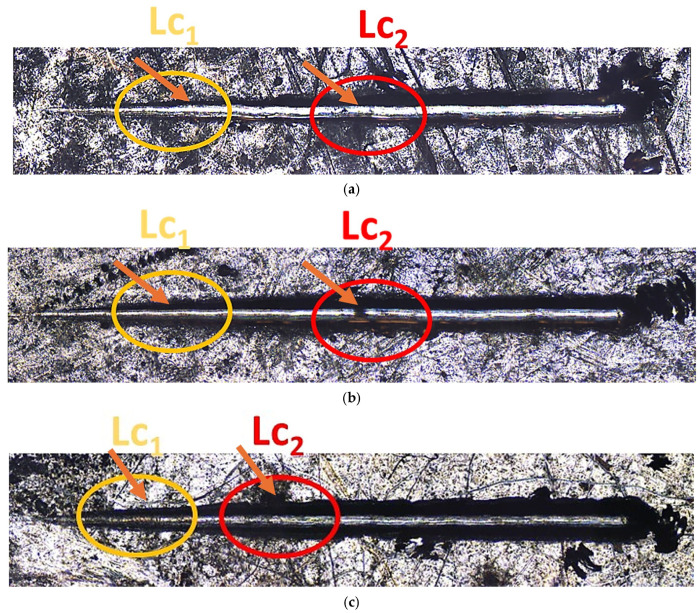
Scratch test images with marked locations of the Lc_1_ and Lc_2_ cracks; (**a**–**c**) sample 1–3, respectively.

**Figure 12:**
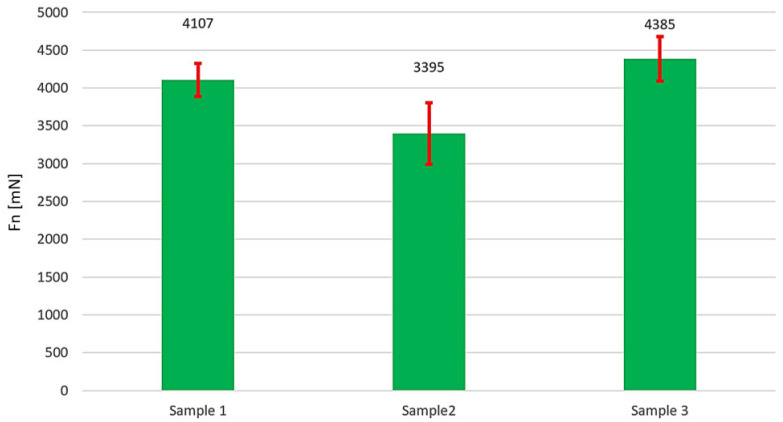
Average values of the critical force for the coating-substrate system for the Lc_1_ crack.

Figure 11 shows photos of scratch marks for all three samples. The images show the locations of coating cracks in the coating-substrate system. Cracks may propagate from the substrate towards the coating or, most often, appear in the coating, directing the crack towards the substrate. The photos were taken using an Anton Paar tribotester equipped with a Rockwell indenter with a rounded tip radius of 200 µm. L_c1_ cohesive cracks and L_c2_ adhesive cracks are marked in the images (Figure 11). After image analysis and measurement of the average value of critical loads, which led to the formation of cohesive cracks, it was observed that the coatings of samples 1 and 3 cracked at a force above 4000 mN, while the coating of sample 2 cracked at 3395 mN (Figure 12). For thinner coatings (samples 1 and 2), cohesive cracks were visible locally in the indenter axis (Figure 11a,b). For the system with the thickest coating (sample 3), from the moment the critical force was exceeded, an avalanche pattern of cracks was visible. These cracks were concentrated along the axis of contact between the indenter and the coating and spread towards the outflow contour (Figure 11c).

**Figure 13:**
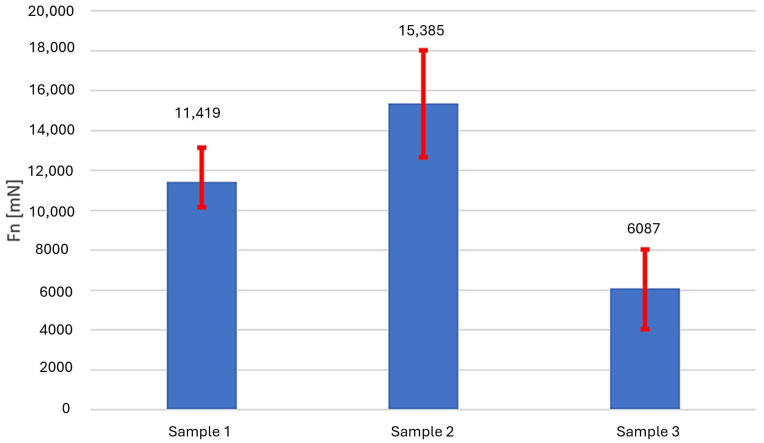
Average values of the critical force for the coating-substrate system for the Lc_2_ crack.

In the further part of the profile, visible locations of L_c2_ adhesion crack formation were marked for all samples. Cracks of this type represent detachment of the coating from the substrate and crumbling of the coating (Figure 11). Image analyses and measurements of the maximum critical force at which L_c2_ adhesive cracks occur showed that the thickest coating cracked and crumbled at a lower critical force than the thinner coatings (Figure 11b and Figure 13). The cracks in the thickest coating were densely arranged as the load increased and were radial in nature. Such an image shows that, under very high loads, thick coatings are susceptible to brittle cracking. In the case of the other two thinner coatings, detachment of the coating from the substrate was visible in the form of larger coating fragments (Figure 11a,b).

**Figure 14:**
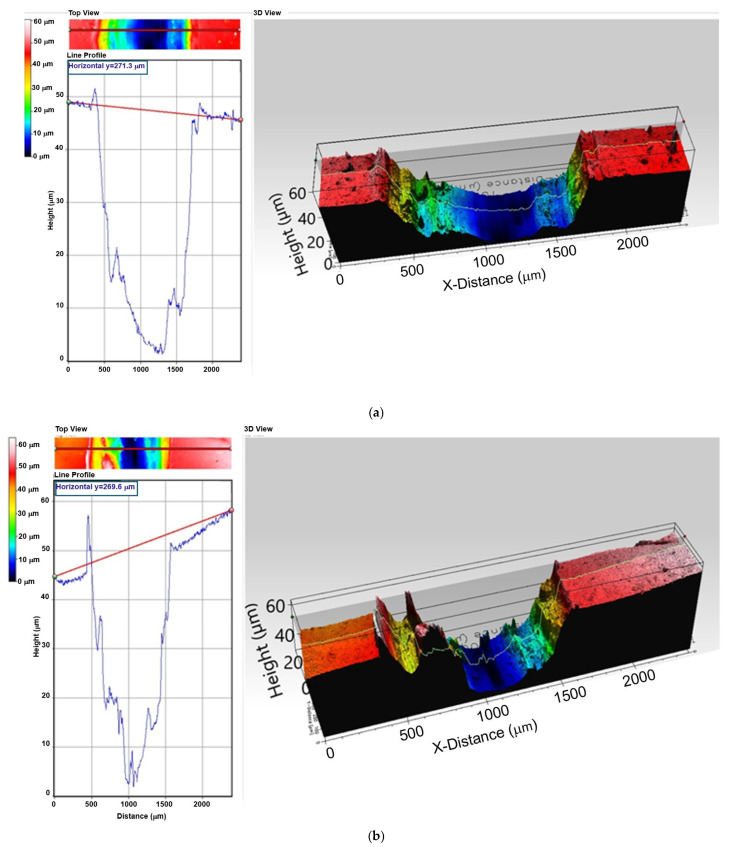
Deformation images of systems with coating (**a**) sample 3 and coating (**b**) sample 2 after the scratch test at the average value of critical loads.

The most frequently observed forms of destruction of the coating–substrate system were analyzed under conditions of elastic deformation and after exceeding the limit at which the coating or substrate underwent plastic deformation. Analysis of the images from the optical profilometer made it possible to observe the curvature of the coating outflow outside the contact zone of the indenter with the coating in samples 2 and 3 (Figure 14). In the case of the coating–substrate system (sample 3), a much larger radius of outflow rounding was visible (Figure 14a). However, for both cases, the elastic nature of the deformations of the coating-substrate system was observed. In the case of a thinner coating, greater system stiffness was visible, which was reflected in the smaller outflow radius (Figure 14b).

**Figure 15:**
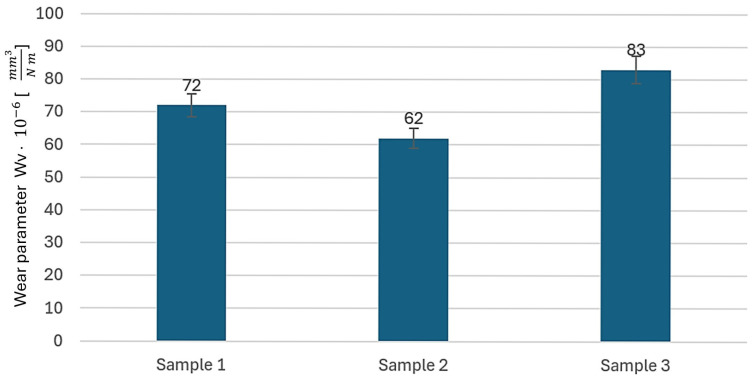
Surface wear rate of the tested samples.

Figure 15 shows the consumption of materials with the corresponding coatings. The average consumption, which represented the ratio of profile–volume measurements to the number of cycles, showed the assumed test result. Thanks to these calculations, it was observed that the thin coatings wore out faster than the thicker ones.

## 4. Conclusions

The conducted microstructural, X-ray diffraction, and abrasive wear resistance studies made it possible to draw the following conclusions:

1. Metallographic observations revealed a layered structure of the zinc coating, consisting of the η, ζ, δ, and Γ phases with different chemical compositions, as confirmed by EDS analysis.
2. The hardness measurement results reflect changes in the phase composition across the tested coatings. The lowest hardness was obtained in the η layer (Zn(Fe)), and the highest in the δ layer (FeZn_10_).
3. The final coating thickness depends directly on the geometry of the grating. The increased coating thickness is associated with sharp edges, over which molten zinc flows more slowly. Zinc remains longer in hard-to-reach areas before draining, leading to local accumulations.
4. The analysis of the scratch test results demonstrates that the critical load values are governed not only by the properties of the coating–substrate system but also by the test parameters themselves. Among these parameters, the loading rate exhibits the greatest influence, as it determines the distribution of contact stresses and affects the onset of both cohesive and adhesive failure mechanisms.
5. The first cohesive cracks and the onset of coating delamination were observed at similar critical load values for all investigated coatings. These results indicate a comparable resistance of the coating systems to damage initiation under progressive loading conditions.
6. However, the microscopic analysis of the scratch tracks revealed differences in the damage evolution mechanisms. In the case of sample 3, which exhibited the greatest coating thickness, adhesive cracks appeared at an earlier stage of damage propagation than in the other coating systems. This behavior may suggest that increased coating thickness promotes stress concentration within the coating–substrate system, thereby accelerating the development of adhesive failure despite the similar critical load values associated with damage initiation.

## Figures and Tables

**Table 1:** Chemical composition of S235JR steel (wt. %).

Content of Elements, wt. %										
C	Mn	Si	P	S	Cu	Cr	Ni	Al	N	Fe
0.1	0.38	0.02	0.018	0.0018	0.03	0.09	0.04	0.03	0.006	balance

**Table 2:** Chemical composition of zinc bath (wt. %).

Zinc Bath Element Content, wt. %								
Pb	Fe	Cd	Cu	Sn	Al	Ni	Bi	Zn
0.34	0.026	0.0007	0.0034	<0.0005	0.0025	0.041	<0.005	balance

**Table 3:** Chemical composition of phases (wt. %), SEM-EDS analysis.

Phase		η		ζ		δ		Γ	
Sample No	Area	Zn	Fe	Zn	Fe	Zn	Fe	Zn	Fe
1	A	100	0	93.5	6.5	90.5	9.5	72.8	27.2
1	B	100	0	93.5	6.5	91.5	8.5	57.4	42.6
2	A	100	0	93.5	6.5	91.7	8.3	70.9	29.1
2	B	100	0	93.9	6.1	91.5	8.5	67.7	32.3
3	A	100	0	93.7	6.3	90.2	9.8	70.2	29.8
3	B	100	0	93.7	6.3	91.3	8.7	67.0	33.0

**Table 4:** Average coating thicknesses (μm).

Phase		η	ζ	δ	Total Thickness
Sample No	Area	Layer Thickness			
1	A	13.9	15.9	10.0	39.8
1	B	15.6	29.9	7.9	53.4
2	A	15.2	24.2	11.6	51
2	B	9.2	30.8	10	50
3	A	16.6	20.0	10.5	47.1
3	B	31.8	53.7	9.5	95

**Table 5:** Microhardness of layers.

Measurement		1 (η)	2 (ζ)	3 (δ)	4 (Steel)
Sample No	Area	HV0.01	HV0.01	HV0.01	HV0.01
1	A	57 ± 3	157 ± 19	259 ± 10	150 ± 5
1	B	52 ± 6	173 ± 14	266 ± 18	171 ± 7
2	A	56 ± 5	112 ± 25	249 ± 12	160 ± 3
2	B	63 ± 7	137 ± 24	267 ± 13	148 ± 6
3	A	78 ± 4	132 ± 17	280 ± 9	151 ± 4
3	B	62 ± 6	123 ± 27	230 ± 7	173 ± 5

## Data Availability

The original contributions presented in this study are included in the article. Further inquiries can be directed to the corresponding authors.
